# Management of acute coronary syndromes in a developing country; time for a paradigm shift? an observational study

**DOI:** 10.1186/s12872-015-0125-y

**Published:** 2015-10-24

**Authors:** Arjuna Medagama, Ruwanthi Bandara, Chinthani De Silva, Manoj Prasanna Galgomuwa

**Affiliations:** Department of Medicine, Faculty of Medicine, University of Peradeniya, Galaha Rd, Peradeniya, 20400 Sri Lanka; Professorial Medical Unit, Teaching Hospital, Peradeniya, Sri Lanka

**Keywords:** Acute coronary syndrome, Sri Lanka, Thrombolysis

## Abstract

**Background:**

There are limited contemporary data on the presentation, management and outcomes of acute coronary syndromes (ACS) in Sri Lanka. We aimed to identify the critical issues that limit optimal management of ACS in Sri Lanka.

**Methods:**

We performed a prospectively observational study of 256 consecutive patients who presented with ACS between November 2011 and May 2012 at a tertiary care general medical unit in Sri Lanka.

**Results:**

We evaluated data on presentation, management, in-hospital mortality, and major adverse cardiovascular events (MACE) of participants. Smoking, alcohol abuse, and obesity were more common in patients with ST elevation myocardial infarction (STEMI) (*P* < 0.05). Discharge diagnoses were STEMI in 32.8 % (84/256) and unstable angina (UA)/non-ST elevation myocardial infarction [NSTEMI] in 67.1 % (172/256) of participants. The median time (IQR) from onset of pain to presentation was 60 (319) minutes for STEMI and 120 (420) for UA/NSTEMI (P = 0.058). A median delay of 240 min was noted in patients who had presented initially to smaller hospitals. Cardiac markers were assessed in only 35 % of participants. In-hospital anti-platelet use was high (>92 %). Only 70.2 % of STEMI patients received fibrinolytic therapy. Fewer than 20 % of patients were received fibrinolytic therapy within 30 min of arrival. Major adverse cardiac events (MACE) were recorded in 11.9 % of subjects with STEMI and 11.6 % of those with UA/NSTEMI (*P* = 0.5). According to logistic regression analysis, body mass index (*P* = 0.045) and duration of diabetes (*P* = 0.03) were significant predictors of in-hospital MACE. On discharge, aspirin, thienopyridine, and statins were prescribed to more than 90 % of patients. Only one patient underwent coronary angiography during the index admission.

**Conclusions:**

Delays in presentation and in initiation of thrombolytic therapy and coronary interventions are key hurdles that need attention to optimize ACS care in Sri Lanka.

## Background

Sri Lanka, a developing Asian country, has a population of 21 million and a rapidly increasing burden of non-communicable diseases. South Asians experience higher fatality rates and premature death attributable to cardiovascular disease (CVD) (deaths occurring at least 10–15 years before expected) than do persons in Western countries [1, 2]. Cardiovascular deaths and disease incidence have increased at an alarming rate in low and middle-income countries [3].

In 2005, the prevalences of hypertension, diabetes, and dysglycemia in Sri Lanka were 20, 11, and 20 %, respectively [4]. Mortality from CVD in Sri Lanka is recognized to be one of the highest causes of death worldwide [5]. Ischemic heart disease (IHD) is the leading cause of death in Sri Lanka, accounting for 27.6 deaths per 100,000 people. Data from 2004–2012 show a steady increase in mortality from IHD in hospitals; in 2012 IHD accounted for 14.4 % of all deaths in hospitals in Sri Lanka [6]. This high mortality rate remains unexplained, but may be attributable in part to a combination of modifiable and non-modifiable cardiovascular risk factors. Previous research has shown that management of CVD in Sri Lanka [7–9], regionally [10], and in developing countries is generally suboptimal compared to Western developed countries [11].

The term acute coronary syndrome (ACS) encompasses a spectrum of coronary artery diseases, including unstable angina (UA), ST-segment-elevation myocardial infarction (STEMI), and non-ST elevation myocardial infarction (NSTEMI). While clinical trials have provided clinicians with evidence for many interventions and medications for ACS, observational studies provide useful information on differences and shortcomings in management practices among countries as well as within different regions of the same country [12, 13].

Treatment with aspirin for patients experiencing ACS and post discharge treatment with aspirin, statins, and blood pressure lowering drugs are cost-effective strategies and considered “best practice” by the World Health Organization [14].

Previous published studies in Sri Lanka have highlighted the following issues in management of ACS: late presentation [8, 9], time delays between presentation and arrival to out-patient departments and wards [9], and less than optimal adherence to current ACS guidelines [7].

The aim of the current study was to gain insights into the following aspects of ACS: epidemiology, presentation, in-ward management, and adherence to current management guidelines, for patients admitted to a tertiary care hospital in Sri Lanka.

## Methods

This prospective observational study was carried out at the Professorial Medical Unit of Teaching Hospital, Peradeniya, Sri Lanka over a 6-month period beginning in November 2011. The hospital is a busy, state-run, tertiary-care institution with nonselective intake of acute patients.

All patients presenting to the out-patients’ department with chest pain within the preceding 24 h and fulfilling ECG criteria for acute STEMI, NSTEMI and UA were included in the study. Such patients were recruited consecutively to avoid selection bias. A diagnosis of STEMI was made in subjects with electrocardiographic (ECG) changes that fulfilled current ECG criteria for the diagnosis of acute STEMI or new-onset left bundle-branch block, and increased serum concentrations of biochemical markers of myocardial necrosis where these measurements were available. Diagnoses of UA and NSTEMI were made in the presence of ischemic type chest pain and ECG changes of ST segment depression more than 1 mm and T wave inversion greater than 1 mm in contiguous leads. Troponin concentrations are not checked routinely at most government hospitals; when required, the test is performed in a private laboratory at the patient’s expense. The distinction between UA and NSTEMI is therefore not routinely made because troponin concentrations are assessed only for patients with uncertain diagnoses or for those who are willing to pay for the test to be performed. The initial diagnosis of ACS was made by a senior house officer or registrar on admission and later confirmed by a consultant physician.

Every patient presenting with chest pain underwent assessment of their history, physical examination, and ECG; those fulfilling the criteria for ACS were included in the present study. Data on pre-hospital and in-ward management were collected from patient records and clarifications were made when necessary by the authors interviewing the patients.

All participants received standard care, that is, their participation in the study did not alter the care they received. Ethical approval for this study was obtained from the Ethics Review Committee of the Faculty of Medicine of the University of Peradeniya, Sri Lanka (2011/EC/22). Informed written consent was obtained from all participants prior to collecting data and was formally recorded.

### Statistical analysis

Continuous variables are presented in means (standard deviation) or median (inter quartile range), when skewed in distribution. Categorical variables are presented as proportions or percentages. ACS types were compared via analysis for variance for continuous variables and the *χ*^2^ test for categorical variables. A two-sided *P* value <0.05 was defined as statistically significant. Univariate and multivariate logistic regression was used to assess possible predictors of death and in-hospital major cardiovascular events (MACE being defined as death, re-infarction, stroke, heart failure, or cardiogenic shock).

## Results

### Study subjects

The study group comprised 256 patients with confirmed ACS, 115 (44.9 %) men and 141 (55.1 %) women. The mean age was 63.2 (STD 11.1) years. Most patients were in the 51–70 year age group (145, 57 %), followed by those aged over 70 years (77, 30 %) and then by those aged less than 50 years (34, 13.3 %). There were no significant age variations between STEMI and UA/NSTEMI patients. The socio-demographic profile of the subjects is summarized in Table 1.

**Table 1:** Patient baseline characteristics according to discharge diagnosis and overall

Variable	STEMI (*n* = 84)	ACS (*n* = 172)	Total (*n* = 256)	*P* value
Sex Male	22 (26.2 %)	93 (54.1 %)	115 (44.9 %)	
Female	62 (73.8 %)	79 (45.9 %)	141 (55.1 %)	
Age (Y)-mean	62.19 (SD = 11.4)	63.7 (SD = 11.1)	63.2 (SD = 11.2)	0.316
Age Strata <50 year	13 (15.5 %)	21 (12.2 %)	34 (13.3 %)	0.564
51–70 year	49 (58.3 %)	96 (55.8 %)	145 (56.6 %)	
>70 year	22 (26.2 %)	55 (31.9 %)	77 (30.1 %)	
Race Sinhala	61 (72.6 %)	151 (87.8 %)	212 (82.8 %)	
Muslim	15 (17.9 %)	17 (9.9 %)	32 (12.5 %)	
Tamil	8 (9.5 %)	4 (2.3 %)	12 (4.7 %)	
Presentation				
Chest pain	75 (89.3 %)	149 (86.6 %)	224 (87.5 %)	0.546
Shortness of breath	1 (1.2 %)	6 (3.5 %)	7 (2.7 %)	0.290
Median time to present (minutes)	60	120	120	0.058
Presenting time >6 h	12 (14.3 %)	34 (19.8 %)	46 (18 %)	0.480
Delay at transfer in minutes (median)	120, IQR = 397.2	240, IQR = 720	180, IQR = 634.8	0.334
Risk Factors				
Coronary artery disease/MI	7 (8.3 %)	46 (26.7 %)	53 (20.7 %)	0.001
Heart Failure	7 (8.3 %)	19 (11.2 %)	26 (10.2 %)	0.059
Stroke	0 (0 %)	7 (4.1 %)	7 (2.8 %)	0.158
Family History Of Cardio Vascular Disease	19 (22.6 %)	32 (18.9 %)	51 (20.1 %)	0.355
History of PCI or CABG	1 (1.2 %)	0 (0 %)	1 (0.4 %)	0.155
Hypertension	26 (31 %)	99 (57.6 %)	125 (48.8 %)	0.001
Dyslipidemia	7 (8.3 %)	12 (7 %)	19 (7.4 %)	0.726
Diabetes Mellitus	27 (32.1 %)	64 (37.2 %)	91 (35.5 %)	0.371
BMI mean	24.63 (SD = 5.287)	23 (SD = 3.687)	23.51 (SD = 4.312)	0.018
Normal BMI (<23 kg/m2)	34 (40.5 %)	86 (50 %)	120 (46.9 %)	
Over Weight (BMI 23–27 kg/m2)	31 (36.9 %)	61 (35.5 %)	92 (35.9 %)	
Obese (BMI >27 kg/m2)	19 (22.6 %)	25 (14.5 %)	44 (17.2 %)	
Waist circumference (mean) in cm	85.61	83.54	84.23	0.092
Current Smoker	17 (20.2 %)	12 (7 %)	29 (11.3 %)	0.001
Alcohol Misuse	23 (27.4 %)	25 (14.5 %)	48 (18.8 %)	0.035

Discharge diagnoses were STEMI in 32.8 % (84/256) and UA/NSTEMI in 67.1 % (172/256). The troponin concentrations of 19 of the 84 STEMI patients were measured and all were positive. Troponin concentrations were measured in only 70 (40.6 %) of the remaining 172 patients with ACS and 42 of those were positive, confirming a diagnosis of NSTEMI. Troponin concentrations were negative in 28 of this group, so they were categorized as having UA. Because a distinction between UA and NSTEMI was not made in most patients in this group, they were considered as having UA/NSTEMI.

Sixty-seven percent of patients came directly to our center, whereas the remainder (33 %) first visited either a general practitioner (GP) or other hospitals in which ACS was not managed. Only 15 % of these patients were transported to our center by ambulance, the remainder had to provide their own transport. The median delay during transfer was 4 h.

The median (interquartile range) delay from symptom onset to hospital admission was shorter for STEMI patients at 60 (319) minutes than for UA/NSTEMI patients at 120 (420) minutes (*P* = 0.058); this difference was not statistically significant. For patients initially presenting to a GP or other hospital for either STEMI or UA/NSTEMI, there was a median delay of 240 (IQR 480) minutes from symptom onset to hospital admission.

Baseline characteristics of the study subjects are shown in Table 1. The body mass index (BMI) and waist circumference of patients with STEMI were higher than those of patients with UA/STEMI. Smoking and alcohol abuse was more common among patients with STEMI than among those with UA/NSTEMI. Generally patients with UA/NSTEMI had a worse medical history and more risk factors than those with STEMI (Table 1).

### Management in hospital and at discharge

Interventions and in-hospital treatments are shown in Table 2. The most commonly used medications were aspirin (95.3 %), clopidogrel (98 %), statins (96.1 %), angiotensin-converting enzyme (ACE)/angiotensin II receptor blockers (ARB) (87 %), and beta blockers (68.7 %) with little differences in the medications administered between patients with STEMI and those with UA/NSTEMI. However, only 16 % those presenting initially to a GP or other hospital received aspirin, compared with 95.3 % at our center (*P* < 0.01). Aspirin was the commonest medication to be withheld, either because of presumed gastric bleeding or epigastric pain. Beta blockers were more commonly given to patients with STEMI (75 %) than to those with UA/NSTEMI (65.7 %, *P* = 0.07) Enoxaparin or unfractionated heparin was given to 87.8 % of patients with UA/NSTEMI and 62 % of those with STEMI.

**Table 2:** Selected in-hospital management, medications, and interventions according to discharge diagnosis

		STEMI (*n* = 84)	ACS (*n* = 172)	Whole population (*n* = 256)	*P* value
Aspirin		(78) 92.9 %	(166) 96.5 %	(244) 95.3 %	0.194
Clopidogel		(82) 97.6 %	(169) 98.3 %	(251) 98.0 %	0.464
Statins		(81) 96.4 %	(165) 95.9 %	(246) 96.1 %	0.987
Beta blockers	Atenolol	(6) 7.1 %	(23) 13.4 %	(29) 11.3 %	0.073
	Carvedilol	(10) 11.9 %	(11) 6.4 %	(21) 8.2 %	
	Metaprolol	(47) 56.0 %	(79) 45.9 %	(126) 49.2 %	
	Total	(63) 75 %	(113) 65.7 %	(176) 68.7 %	
ACEI, ARB	Captopril	(2) 2.4 %	(21) 12.2 %	(23) 9.0 %	0.946
	Enalapril	(66) 78.6 %	(101) 58.7 %	(167) 65.2 %	
	Losartan	(5) 6 %	(28) 16.3 %	(33) 12.9 %	
	Total	(73) 87 %	(150) 87.2 %	(223) 87.1 %	
Nitrates		(31) 36.9 %	(72) 41.9 %	(103) 40.9 %	0.145
Heparin		(20) 23.8 %	(28) 16.3 %	(48) 18.8 %	0.167
Enoxaparin		(52) 61.9 %	(151) 87.8 %	(203) 79.3 %	0.001
Diuretics		(18) 21.4 %	(25) 14.6 %	(43) 16.9 %	0.209
Troponin done		19 (22.6 %)	70 (41.7 %)	89 (35.3 %)	0.004
Troponin positive in		19 (100 %)	42 (60 %)	61 (23.8 %)	-
Fibrinolysis		(59) 72.8 %	(0) 0 %	-	-
Door to needle time: median (IQR) min		64 (55)			
<30 min		(10) 16.9 %			
30–60 min		(18) 30.5 %			
>60 min		(31) 52.5 %			
Transferred to Ward		36 (43.3 %)	129 (78.2 %)	165 (67.6 %)	0.001
HDU		43 (58.1 %)	31 (19.3 %)	74 (30.3 %)	0.001
ICU		4 (4.8 %)	1 (0.6 %)	5 (2 %)	0.028
MACE		10 (11.9 %)	20 (11.6 %)	30 (11.7 %)	0.948
Echocardiography performed in		71 (84.5 %)	164 (95.3 %)	235 (91.8 %)	0.003
EF mean (%)		43.38 (SD 18.7)	52.65 (SD 13.6)	49.65 (SD 16.0)	0.001
Stress Test planned on discharge		19 (24.1 %)	44 (26.5 %)	63 (25.7 %)	0.866
Referred for	PCI	1 (1.2 %)	0 (0 %)	1 (0.39 %)	
	Angiogram	9 (11.5 %)	11 (6.9 %)	20 (8.4 %)	
	CABG	0 (0 %)	1 (0.6 %)	1 (0.4 %)	
Mean duration of hospital stay		5.12 (SD = 1.430)	4.33 (SD = 2.089)	4.57 (SD = 1.946)	0.001

Fifty-nine patients (70.2 %) with STEMI underwent fibrinolytic therapy with streptokinase. Thirteen of the 84 (15.4%) patients with STEMI were not eligible for thrombolytic therapy because of their late presentation. The remaining 12 patients were eligible on the basis of arrival time; however, three of these (3.7 %) had absolute or relative contraindications to thrombolytic therapy; the reasons for the remaining nine (11.1 %) eligible patients not undergoing this treatment were unknown. Median door-to-needle time was 64 (IQR 55) minutes. Only 10 patients (16.9 %) received thrombolytic therapy within 30 min, 28 (47.4 %) within 60 min, and 39 (66 %) within 90 min. Twenty (33.9 %) received thrombolytic therapy more than 90 min after arriving at the hospital.

Mean duration of in-hospital stay was 5.12 (1.4) days for patients with STEMI, which was greater than the mean of 4.3 (2.0) days for patients with UA/NSTEMI (*P* < 0.01). Overall, only 32.3 % of patients were managed in an intensive care or high dependency unit.

There were no reported deaths in patients with STEMI and two in those with UA/NSTEMI. Thirty (11.7 %) patients developed MACE, which was defined as death, re-infarction, stroke, heart failure, or cardiogenic shock occurring during their hospital stays (Table 2). One patient with STEMI had major non-fatal bleeding.

During the index admission, 84.5 % of patients with STEMI and 95.3 % of those with UA/NSTEMI had transthoracic echocardiography performed. The mean ejection fraction of patients with STEMI was 43.38 % (18.7), which was significantly lower than the mean ejection fraction 52.6 % (13.6) for those with UA/STEMI (*P* < 0.001). Coronary angiography was performed on only one patient during the index admission. At discharge, coronary angiography was planned for 20 patients (8.4 %) and cardiac stress testing for 63 (25.7 %).

At discharge, medications prescribed included aspirin (94.8 %), clopidogrel (97.8 %), statins (96.1 %), ACEI/ARB (82 %), and beta-blockers (72.6 %).

### Predictors of in-hospital mortality and morbidity

During the index admission, MACE occurred in 30 (11.7 %) of patients. Logistic regression showed BMI (*P* = 0.045) and duration of diabetes (*P* = 0.03) were significant predictors of MACE. Patients presenting with STEMI did not have a higher risk of MACE than UA/STEMI patients. Time to presentation, presence of hypertension, diabetes, and previous cardiac disease were not significant predictors of MACE.

## Discussion

In this study, ACS was diagnosed with reasonable accuracy using clinical and ECG criteria. Troponin concentrations were measured only in a minority of patients because of the non-availability of this test in the state sector and its high cost in private-sector laboratories.

Teaching Hospital, Peradeniya is one of the two largest tertiary-care institutions in the area; many smaller hospitals redirect patients with ACS to our unit. Thus, we believe that our sample can be considered representative of patients with ACS in this region.

The management of patients presenting with ACS in Sri Lanka has not been well documented. Previous studies have explored the time delay to initiation of thrombolytic therapy and adherence to guidelines [7–9]. Sri Lanka still relies heavily on medical management of ACS as opposed to primary coronary intervention. At present, few centers have commenced treating patients with primary percutaneous coronary intervention (PCI) [3].

There was no significant difference in age distribution between patients with STEMI and those with UA/NSTEMI, the majority of patients were in the 51–70 year age range in both groups. Patients with STEMI tended to have fewer risk factors and less frequently had a history of previous cardiac disease; they were however, more often smokers and had higher BMI. Hypertension and previous cardiac disease were notably more prevalent in the UA/NSTEMI group. The prevalence of risk factors among patients in Sri Lanka with ACS has not been reported previously. The prevalences of hypertension, diabetes, and previous cardiac disease (48.8, 35.5 and 20.7 %, respectively) found in this study are comparable to those reported for India (48.4, 37.6 and 14.2 %, respectively) [10]. However, the Acute Coronary Events – a Multinational Survey of Current Management Strategies (ACCESS) investigators reported a higher prevalence of hypertension (STEMI 78 %, NSTE-ACS 87 %) among ACS patients in other developing countries compared to Sri Lanka and India [11].

The types of ACS diagnosed in the current study were broadly similar to those reported for other observational studies conducted locally and globally. Rajapakse et al. [7] reported that 33.6 % of ACS patients in Sri Lanka had STEMI. Mohanan and colleagues reported a similar rate of 37 % in India. The ACCESS group of investigators reported that in developing countries 46 % of ACS patients had STEMI and 54 % NSTEMI-ACS. Our finding of 32.8 % for STEMI is marginally lower than previously reported figures.

The median time delay from symptom onset to presentation was shorter in patients with STEMI than in those with UA/NSTEMI (60 min vs. 120 min, *P* = 0.058); this difference was not statistically significant. Previously reported median pre-hospital delays for Sri Lankan patients with STEMI were 130 [8] and 720 min [9]; our results show a considerable improvement over these previous findings. The median in-hospital delay in admitting patients to an acute care unit was 5 min in the current study compared with previously reported figures of 70 [8] and 20 min [9]. Pre-hospital delays were longer for those who sought pre-hospital care than for those who presented directly to our institution. In previous studies, the reported average symptom onset to treatment time has ranged from 1.5–6 h globally [15]. The ACCESS study reported median delays of 4.0 and 6.0 h for patients with STEMI and NSTEMI-ACS, respectively [11]. The European Society of Cardiology has identified that the pre-hospital period is the most critical phase for reducing mortality and reiterated that efforts should be made to shorten this delay [16].

The overall use of aspirin, thienopyridines, and statins on admission, in-ward and at discharge were satisfactory [greater than 92 % (Table 2)]. The use of these medications did not differ significantly between patients with STEMI and those with UA/NSTEMI. Beta blockers were underutilized at admission (30.1 %), during hospital stay (68.7 %), and at discharge (72.6 %). The World Health Organization considers the use of aspirin, statins, and blood pressure-reducing agents post discharge to be a cost-effective strategy for patients with ACS; these medications are considered *best buys* [14].

In the present study, 83 % of eligible patients received thrombolytic therapy, a result consistent with the findings of Rajapakse et al., who reported that thrombolytic therapy was administered in 84.6 % of patients with STEMI [7]. Both figures show a tremendous improvement from the 17 % reported in Sri Lanka in 1999 [9]. However, in our study, a considerable proportion of patients with STEMI (30 %) received neither thrombolytic therapy nor PCI. Reported rates of thrombolytic therapy are 24.7 % in Kerala, India [10] and 39 % in the ACCESS study, which was performed in developing countries besides India and Sri Lanka [11]. However the overall rates of reperfusion therapy (PCI and coronary artery bypass graft surgery [CABG]) in both these studies were considerably higher (14 and 43.8 %, respectively) than in the present study.

Only 1 % of our study subjects underwent an interventional reperfusion procedure during the index admission. This largely reflects the current management of patients with ACS in the state sector, which caters to over 90 % of the population. At present, primary PCI is being introduced in few state-run cardiology units in the country. This probably reflects the limited resources of a state run, free health care system prevalent in Sri Lanka. However, coronary angiography and primary PCI are available in Sri Lanka in fee levying centers [3]. Rates of coronary angiography rates in Kerala, India were 19.6 and 18.6 % for patients with STEMI and NSTEMI-ACS, respectively, [10], 56 and 59 %, respectively, in the ACCESS study [11] and 62–63 and 70–80 %, respectively, in the second Euro Heart Survey (EHS-ACS-II) [17] and Global Registry of Acute Coronary Events (GRACE) [18]. The rates of PCI in the ACCESS, EHS-ACS-II, and GRACE studies for NSTEMI/ACS and STEMI were 31 and 41 %, 35 and 58 %, and 47 and 64 % respectively [8].

There is global evidence for favoring early coronary angiography and intervention following ACS [19, 20]. A recent meta-analysis has shown that complete revascularization (as opposed to culprit-only revascularization) during primary PCI may have beneficial outcomes at one year [21]. This largely reflects the non-critical atherosclerotic disease burden in coronary arteries, which unfortunately is not addressed by medical revascularization. We believe that the available evidence strongly favors an urgent transition from medical revascularization to PCI in Sri Lanka.

Median door-to-needle time in our study was 64 min; the mean of 96 min was attributable to late initiation of thrombolytic therapy in some patients. The recommended door to needle time of 30 min was achieved in only 16.9 % of study subjects. A previous study reported a median door-to-needle time of 70 min [8]. In Malaysia the median door-to-needle time is reportedly 48–54 min [22]. In India, the door to needle time exceeded 30 min in only 31 % of patients with STEMI [10].

During the index admission, MACE was observed in 11.7 % of patients; this incidence did not differ significantly between STEMI and UA/NSTEMI groups. Two deaths occurred in the UA/NSTEMI group and none in the STEMI group; however, the small sample size prevents drawing any definite conclusions from these findings. The in-hospital mortality rates for patients with STEMI is reportedly 8.2 % in the Kerala registry in India, 7 % in the GRACE study and 4.3 % in the ACTION study [23]. The in-hospital mortality for patients with UA/NSTEMI in our study (1.1 %) is low compared with reported mortality rates of 1.8 % in India [10], 3 % in the Euro heart survey ACS II [17], and 3.9 % in the ACTION study [23] for patients with NSTEMI.

Because Sri Lanka does not currently have a cardiac registry, the outcomes of ACS following the index admission are not known. This study attempts to highlight this deficiency and express the urgent need for better record keeping and registration of ACS patients. With the phased-in introduction of primary PCI in selected centers, it would be interesting to compare treatment and outcomes of ACS a few years in the future, just as previous studies [7–9] have paved the way for the current comparisons.

### Implications for quality improvement

Higher BMI and duration of diabetes were significant predictors of in-hospital MACE in the current study. Although we failed to demonstrate time to treatment, and door to needle time as significant predictors of MACE, previous studies have shown that these factors are significant predictors of adverse cardiac outcomes [10]. However, delayed presentation (STEMI, median 60 min and UA/NSTEMI, median 120 min) remains a critical factor in the deployment of effective treatment. Late presentation resulted in 12.3 % of patients with STEMI not receiving fibrinolytic therapy.

Transfer of patients between GPs, smaller hospitals, and ACS treatment facilities remain a hurdle in Sri Lanka’s emergency response system. The observed median delay of 240 min is considerable and largely attributable to most patients having to find their own transport. Educating the public on early warning symptoms, streamlining emergency services at smaller hospitals and GP centers, and developing outreach and emergency cardiac response teams are critical for improving cardiac services in Sri Lanka.

The emergency care provided was minimal in smaller hospitals and at GP practices; only 16 % of patients with ACS receiving aspirin or a thienopyridine. Furthermore, only 16.9 % of patients with STEMI received thrombolytic therapy within the recommended 30 min of admission. Fibrinolytic therapy was not administered to 11 % of eligible patients for unknown reasons, implying both deficiencies in cardiac response team training and poor record keeping.

At the time of this study, PCI was heavily underutilized in the management of ACS in Sri Lanka. Over the last 3 years, significant efforts have been taken to train health workers and increase the use of PCI in treating ACS. Although the use of aspirin, thienopyridines, and statins was satisfactory, beta blocker use was suboptimal in our study sample. Poor utilization of beta blockers has been reported in earlier studies [7] and clearly needs to be addressed.

### Limitations

This study is the first to compare demographic characteristics, presentation and management of patients with STEMI and NSTEMI/UA at a tertiary care center in Sri Lanka. Because of limited resources, cardiac markers were not measured in all patients, preventing further characterization of the NSTEMI/UA group. The relatively small sample size of 256 patients and the absence of post discharge follow up are additional limitations of this study.

## Conclusions

The use of aspirin, thienopyridines, and statins on admission, in ward, and on discharge was satisfactory. Only 72.8 % of STEMI patients received thrombolytic therapy and there was significant delay in initiating fibrinolysis. Emergency care at smaller hospitals was suboptimal and transfer of patients between hospitals was unsafe. PCI was minimally utilized at presentation and thereafter. Instituting safe and effective patient transfer to hospitals, improving the training of first contact doctors, and increasing the use of current PCI practices are key areas that need to be urgently addressed.

## Abbreviations

ACCESS: Acute Coronary Events**—**Multinational Survey of Current Management Strategies
ACE: Angiotensin-converting enzyme
ACS: Acute coronary syndrome
ARB: Angiotensin II receptor blocker
BMI: Body mass index
CVD: Cardiovascular disease
EHS-ACS: Second Euro Heart Survey
GP: General practitioner
GRACE: Global Registry of Acute Coronary Events
IHD: Ischemic heart disease
MACE: Major adverse cardiac events
STEMI: ST segment elevation myocardial infarction
NSTEMI: Non ST segment elevation myocardial infarction
PCI: Percutaneous coronary intervention
UA: Unstable angina
